# Texas Fever and Ticks

**Published:** 1892-04

**Authors:** B. A. Rogers

**Affiliations:** Georgetown


For Daniel's Texas Medical Journal.

### TEXAS FEVER AND TICKS.

BY B. A. ROGERS, M. D., GEORGETOWN.

MY ATTENTION having been called, from the office of publication of DANIEL'S TEXAS MEDICAL JOURNAL to its issue of March (No. 9) in which its columns have been used for the manufacture of a little cheap wit at the supposed expense of myself, the Live Stock Convention and the Agricultural Department of the United States, I write in answer to ask the loan of sufficient space in its next number for a quotation from the report or the Secretary of Agriculture for A. D. 1890, recently issued. Embodied in the General Report is a report from Prof. D. E. Salmon, Chief of the Bureau of Animal Industry, touching a long series of experimental investigation that have been going on for a number of years in his department for the determination of the character and causes of the so-called Texas fever in Northern cattle.

These investigations have been carried on at the government experimental station near Washington, D. C.

I quote as to the work in 1890: "The investigations into the nature and causes of Texas fever have been busily pushed during the summer of 1890, and some very important advances have been made which are destined to be of great practical importance . . During the summer some fifty-three native animals, distributed around in various experimental enclosures at the station, received more or less careful attention . . . ."

"The work of the summer confirmed that done in the previous summers."

Under the head of "THE RELATION OF TICKS TO TEXAS CATTLE FEVER" the report says: "While the investigations into the nature of this disease were going on, equally impo:tant work was carried on at the experimental station on the external characters of the disease."

"It is well known to those who have come in contact with Southern cattle in summer that they are affected with the so-called cattle-tick, a pest belonging to the class *Arachnoidea* and to the family *Ixodidæ.*

"These ticks are carried North with cattle during the warm season of the year.

"When fully matured they drop off from the Southern animal, lay their eggs in the ground and perish. The young ticks are hatched within fifteen to thirty days after the eggs are laid, and at once get upon the cattle, when they become mature within twenty to thirty days, to again drop off, lay their eggs and die. This process goes on continually until the cold weather comes.

"At various times and different parts of the country, it has been suggested that the ticks were the cause of Texas fever in Northern cattle. This inference was undoubtedly suggested by the fact that nearly all cattle that die of Texas fever are observed to have these ticks, of various sizes, attached to the skin. Moreover, the diesase only makes its appearance after the young ticks have attached to cattle. Though this is purely a *post hoc, propter hoc* inference, it is nevertheless true, as the experiments to be recorded will amply prove.

"During the summer of 1889, Dr. F. L. Kilborne, in arranging the various inclosures at the experiment station for the exposure of native cattle to the infection of Texas fever, conceived the happy idea of testing this popular theory of the relation of ticks to the disease. This he did by placing Southern (North Carolina) cattle with native cattle in the same inclosure, and picking the ticks from the Southern stock as soon as they grew large enough to be detected on the skin. This prevented any ticks from maturing, and infecting the pasture with their eggs, and hence prevented any ticks from infesting native cattle subsequently; at the same time, in another inclosure, the ticks were left on the Southern cattle. The natives in the latter field died of Texas fever; those in the former did not show any signs of the disease.

"Another experiment was made in September in the same manner, by preparing three fields, one with Southern cattle and ticks, a second with Southern cattle from which the ticks were removed, and a third over which adult ticks had been scattered. The result was equally positive. In the first field no natives died, but careful examination of the blood by the writer showed Texas fever in an unmistakable manner. In the 'tick' field one animal died of Texas fever, and the examination of the blood showed that most other natives in the field were sick. In the third field, containing Southern cattle without ticks, no disease could be detected.

"These two tests pointed directly to ticks being, in some way, the cause of Texas fever. At the same time, it was thought best to confirm these results by further experiments during the present year, before other agencies could be eliminated. The immediate inference was that the ticks infest the pastures, and that in some unexplained manner infection finds its way into the body of susceptible cattle. The preliminary conclusions deducible from the work of 1888 and 1889 can be formulated as follows:

"1. Texas fever is a disease not caused by bacteria. Its nature can not be understood by supposing a simple transfer of bacteria from Southern cattle to pastures, and from pastures to Northern cattle.

"2. The cause is very probably a protozoon, with a more complex life history, living for a time with the red corpuscles of infected animals.

"3. Southern cattle without ticks can not infect a pasture.

"4. Ticks alone scattered over a pasture will produce the disease.

"The work of 1890 was planned to conform or refute these preliminary conclusions, and to furnish additional information.

"The fields were arranged as before. One contained North Carolina cattle with ticks; a second, Texas cattle with ticks; a third, North Carolina cattle without ticks; a fourth, ticks only; and a fifth, soil from the pastures of infected North Carolina farms."

"The results confirmed those of last year. The first arrival to die was in a 'tick' field containing no Southern cattle. No disease appeared in the soil field. Unfortunately, owing to the limited space of ground at our disposal, and its barren, rolling character, ticks or eggs were washed during the very heavy rains of the summer from the tick field into the field containing Southern cattle without ticks, although a wide lane intervened. The natives of this field thereupon all died of Texas fever. At the autopsy of these cases ticks were found attached to their skin in abundance."

"There seemed to be but one inference to be drawn from the facts, and that is that the presence of young ticks is in some way directly associated with the disease.

"It requires from forty to sixty days for the matured ticks to drop from the Southern cattle, and the eggs laid by them to develop into young ticks. After that period young ticks are present on the pastures until they are destroyed by the cold, or until the cold interferes with the embryo in the egg. In other words, the period of incubation is explained without very much difficulty by the life history of the tick."

The report goes on to give various other experiments in the same line, and with the same results, and adds: "The evidence accumulated thus far, seems to favor very strongly the dictum: no ticks, no Texas fever." (See pages 105 to 111 inclusive.)

The report makes it clear that Texas cattle, though communicating the disease by their ticks, are themselves healthy; that it is a disease of the blood in Northern cattle, and that cattle do not take the disease from contact with each other, but from contact with small ticks.

If this matter is of sufficient importance to occupy the attention of the bureau of animal industry of the United States for years, is it unworthy the thought of the Texas Live Stock Convention, or the experiment station at Bryan?

Does the Editor now see where the laugh comes in.

